# Tetra­kis(μ-2-methyl­benzoato)bis­[(2-methyl­benzoic acid)copper(II)]

**DOI:** 10.1107/S1600536808006661

**Published:** 2008-03-14

**Authors:** Abraham C. Sunil, Barend C. B. Bezuidenhoudt, J. Marthinus Janse van Rensburg

**Affiliations:** a Department of Chemistry, University of the Free State, PO Box 339, Bloemfontein 9300, South Africa

## Abstract

In the title centrosymmetric dinuclear compound, [Cu_2_(C_8_H_7_O_2_)_4_(C_8_H_8_O_2_)_2_], four *o*-toluate anions form a cage around two Cu atoms in a *syn*–*syn* configuration. Two more *o*-toluic acid mol­ecules are apically bonded to the Cu atoms, which show a square-pyramidal coordination geometry. The acid H atoms are hydrogen bonded to the cage carboxyl O atoms [O⋯O = 2.660 (2) Å]. The mol­ecular packing forms a puckered pseudo-hexa­gonal close-packed layer in the (*h*00) plane, with soft inter­molecular H⋯H contacts (2.46–2.58 Å).

## Related literature

For the synthesis of aromatic carboxylic acids, see: Kaeding (1967 ▶). For tetra­kis(μ_2_-2-fluoro­benzoato)bis­(2-fluoro­benzoic acid)dicopper(II), see: Valach *et al.* (2000 ▶), For tetra­kis(μ_2_-benzoato)bis­(2-fluoro­benzoic acid)dicopper(II), see: Kawata *et al.* (1992 ▶). 
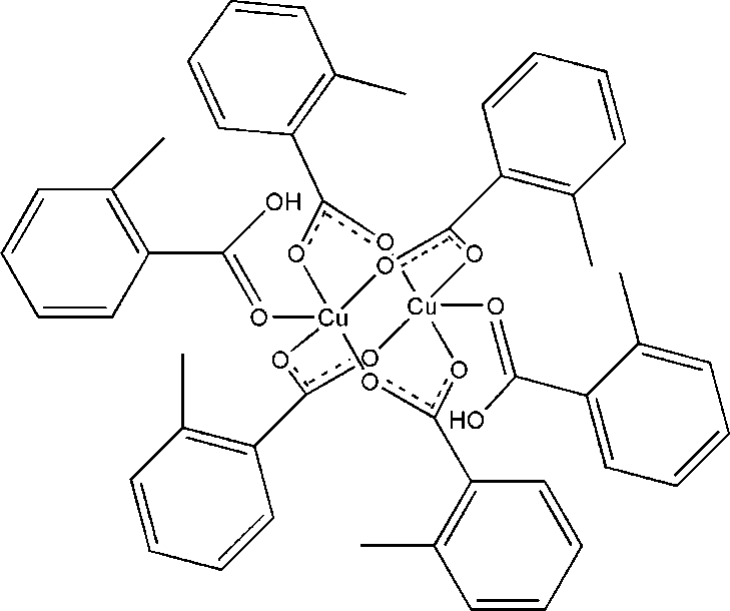


## Experimental

### 

#### Crystal data

[Cu_2_(C_8_H_7_O_2_)_4_(C_8_H_8_O_2_)_2_]
*M* *_r_* = 939.96
Triclinic, 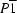

*a* = 10.530 (3) Å
*b* = 10.579 (3) Å
*c* = 10.773 (4) Å
α = 109.248 (2)°
β = 93.156 (2)°
γ = 106.287 (2)°
*V* = 1073.0 (6) Å^3^
*Z* = 1
Mo *K*α radiation
μ = 1.06 mm^−1^
*T* = 100 (2) K
0.25 × 0.08 × 0.06 mm

#### Data collection

Bruker X8 APEXII diffractometer
Absorption correction: multi-scan (*SADABS*; Bruker, 2004 ▶b) *T*_min_ = 0.778, *T*_max_ = 0.939
17497 measured reflections
5138 independent reflections
4668 reflections with *I* > 2σ(*I*)
*R*_int_ = 0.025

#### Refinement

*R*[*F* ^2^ > 2σ(*F* ^2^)] = 0.026
*wR*(*F* ^2^) = 0.065
*S* = 1.05
5138 reflections
284 parameters
H-atom parameters constrained
Δρ_max_ = 0.41 e Å^−3^
Δρ_min_ = −0.33 e Å^−3^

### 

Data collection: *APEX2* (Bruker, 2005 ▶); cell refinement: *SAINT-Plus* (Bruker, 2004 ▶); data reduction: *SAINT-Plus* and *XPREP* (Bruker, 2004 ▶); program(s) used to solve structure: *SIR97* (Altomare *et al.*, 1999 ▶); program(s) used to refine structure: *SHELXL97* (Sheldrick, 2008 ▶); molecular graphics: *DIAMOND* (Brandenburg & Putz, 2005 ▶); software used to prepare material for publication: *WinGX* (Farrugia, 1999 ▶).

## Supplementary Material

Crystal structure: contains datablocks global, I. DOI: 10.1107/S1600536808006661/ng2426sup1.cif

Structure factors: contains datablocks I. DOI: 10.1107/S1600536808006661/ng2426Isup2.hkl

Additional supplementary materials:  crystallographic information; 3D view; checkCIF report

## Figures and Tables

**Table 1:** Selected bond lengths (Å)

Cu1—O21^i^	1.9402 (12)
Cu1—O11	1.9559 (12)
Cu1—O12	1.9585 (13)
Cu1—O22^i^	1.9900 (13)
Cu1—O31	2.1622 (13)
Cu1⋯Cu1^i^	2.5780 (9)

Symmetry code: (i) 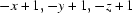.

**Table 2:** Hydrogen-bond geometry (Å, °)

*D*—H⋯*A*	*D*—H	H⋯*A*	*D*⋯*A*	*D*—H⋯*A*
O32—H32⋯O22^i^	0.82	1.85	2.6604 (18)	168
C16—H16⋯O21	0.93	2.39	2.721 (2)	101

Symmetry code: (i) 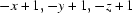.

**Fig. 1.**
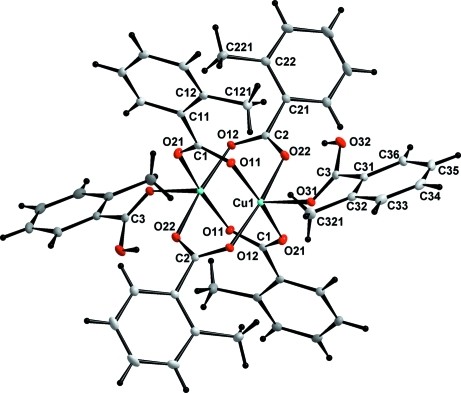
A view of (I) showing the atom-numbering scheme with displacement ellipsoids at the 30% probability level, non labelled atoms are symmetric equivalents. For the phenyl C-atoms, the first digit indicates ring number and the second digit the position of the atom in the ring.

**Fig. 2.**
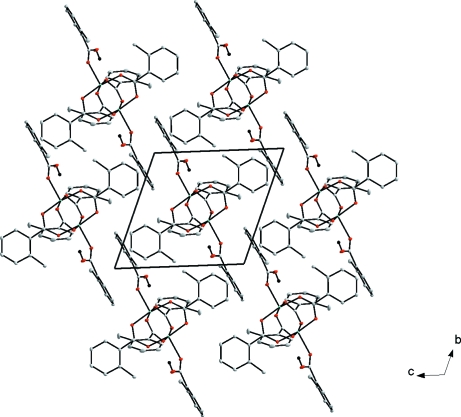
Indication of pseudo-hexagonal close packing along the b axis.

## supplementary crystallographic information

### Comment

In our endeavours to produce phenols from aromatic carboxylic acids, we came across work by Kaeding (1967). In order to verify the reaction sequence as proposed by Kaeding, we prepared the copper salt of *o*-toluic acid and obtained single crystals.

The title compound, (I), [Cu_2_(C_8_H_7_O_2_)_4_(C_8_H_8_O_2_)_2_] crystallized with molecular symmetry -1 (Fig.1). The title compound exhibits a cage-like structure. Four *o*-toluic anionic ligands form a cage around two Cu-atoms in a *syn*-*syn* configuration. Two more carboxylic acid are apically bonded to the Cu-atoms. The acid protons are hydrogen bonded to the cage carboxylate O atoms, O32—H32···O22 = 167.6° and O32···O22 = 2.6604 (18) Å. Another intra-molecular H···H short contact is present at C16···O21 with C16—H16···O21 = 100.9° and C16···O21 = 2.721 (2) Å.

Comparing the Van der Waals radii of copper, 2.32 Å, and the metallic Cu—Cu bond length, 2.55 Å, to the Cu—Cu separation in (I), 2.5780 (9) Å, one would expect the presence of weak orbital interaction. The Cu—O bond lengths of the cage carboxylates vary between 1.9402 (12) - 1.9900 (13) Å. The Cu—O bond distances to the adducted acid molecules show a *ca* 0.2 Å increase. Each Cu atom is displaced from the basal plane of the four caged carboxylates by 0.171 Å, towards the apical O atom. Compound (I) forms a puckered pseudo-hexagonal close packed layer in the (h 0 0) plane, with soft inter-molecular H···H contacts, 2.457–2.580 Å (Fig.2).

### Experimental

The complex [Cu_2_(C_8_H_7_O_2_)_4_(C_8_H_8_O_2_)_2_] was prepared by heating *o*-toluic acid (2.52 g, 18.5 mmol), copper carbonate (0.73 g, 3.3 mmol) and magnesium oxide (0.19 g, 4.68 mmol) under reflux, in toluene (30 ml) for 24 h. The product was extacted and crystallized from diethyl ether to yield a blue crystalline solid. (Yield: 84%)

### Refinement

Hydrogen atoms were placed in calculated positions, and they ride on the parent C-atoms, with U set to 1.2 to 1.5 times *U*_eq_(C).

### Crystal data

**Table:**

[Cu_2_(C_8_H_7_O_2_)_4_(C_8_H_8_O_2_)_2_]	*Z* = 1
*M**_r_* = 939.96	*F*_000_ = 486
Triclinic, *P*1	*D*_x_ = 1.455 Mg m^−^^3^
Hall symbol: -P 1	Mo *K*α radiation λ = 0.71069 Å
*a* = 10.530 (3) Å	Cell parameters from 8974 reflections
*b* = 10.579 (3) Å	θ = 2.6–28.3º
*c* = 10.773 (4) Å	µ = 1.06 mm^−^^1^
α = 109.248 (2)º	*T* = 100 (2) K
β = 93.156 (2)º	Needle, blue
γ = 106.287 (2)º	0.25 × 0.08 × 0.06 mm
*V* = 1073.0 (6) Å^3^	

### Data collection

**Table:**

Bruker X8 APEXII 4K KappaCCD diffractometer	4668 reflections with *I* > 2σ(*I*)
Monochromator: graphite	*R*_int_ = 0.026
*T* = 100(2) K	θ_max_ = 28º
ω and φ scans	θ_min_ = 2.2º
Absorption correction: multi-scan(SADABS; Bruker, 2004b)	*h* = −13→13
*T*_min_ = 0.778, *T*_max_ = 0.939	*k* = −13→13
17497 measured reflections	*l* = −14→14
5138 independent reflections	

### Refinement

**Table:**

Refinement on *F*^2^	H-atom parameters constrained
Least-squares matrix: full	*w* = 1/[σ^2^(*F*_o_^2^) + (0.024*P*)^2^ + 0.7431*P*] where *P* = (*F*_o_^2^ + 2*F*_c_^2^)/3
*R*[*F*^2^ > 2σ(*F*^2^)] = 0.026	(Δ/σ)_max_ = 0.001
*wR*(*F*^2^) = 0.065	Δρ_max_ = 0.41 e Å^−^^3^
*S* = 1.05	Δρ_min_ = −0.33 e Å^−^^3^
5138 reflections	Extinction correction: none
284 parameters	

### Special details

**Table:**

Experimental. The intensity data was collected on a Bruker X8 Apex II 4 K Kappa CCD diffractometer using an exposure time of 30 s/frame. The 1757 frames were collected with a frame width of 0.5° covering up to θ = 28° with 99.3% completeness accomplished.
Geometry. All e.s.d.'s (except the e.s.d. in the dihedral angle between two l.s. planes) are estimated using the full covariance matrix. The cell e.s.d.'s are taken into account individually in the estimation of e.s.d.'s in distances, angles and torsion angles; correlations between e.s.d.'s in cell parameters are only used when they are defined by crystal symmetry. An approximate (isotropic) treatment of cell e.s.d.'s is used for estimating e.s.d.'s involving l.s. planes.

### Fractional atomic coordinates and isotropic or equivalent isotropic displacement parameters (Å^2^)

**Table:**

	*x*	*y*	*z*	*U*_iso_*/*U*_eq_	
Cu1	0.444832 (17)	0.389154 (17)	0.395103 (17)	0.01027 (6)	
O32	0.51055 (11)	0.07974 (12)	0.26534 (12)	0.0217 (2)	
H32	0.5525	0.1594	0.3175	0.033*	
O31	0.36065 (11)	0.18596 (11)	0.23619 (10)	0.0154 (2)	
O21	0.47477 (11)	0.50089 (11)	0.70973 (10)	0.0175 (2)	
O22	0.38457 (10)	0.65099 (11)	0.57571 (11)	0.0168 (2)	
O11	0.38005 (11)	0.30802 (11)	0.52758 (10)	0.0157 (2)	
O12	0.29312 (10)	0.46128 (11)	0.39073 (10)	0.0150 (2)	
C16	0.36586 (15)	0.36861 (16)	0.87485 (15)	0.0161 (3)	
H16	0.3999	0.4666	0.9049	0.019*	
C31	0.31192 (15)	−0.05882 (15)	0.11267 (14)	0.0142 (3)	
C11	0.35899 (14)	0.29021 (15)	0.74017 (14)	0.0126 (3)	
C36	0.37677 (17)	−0.14773 (16)	0.03537 (16)	0.0189 (3)	
H36	0.4698	−0.1226	0.0517	0.023*	
C25	0.00903 (16)	0.72093 (17)	0.56202 (18)	0.0235 (3)	
H25	−0.0326	0.7479	0.6358	0.028*	
C321	0.09555 (16)	−0.00753 (18)	0.17572 (17)	0.0229 (3)	
H32A	0.0954	0.0674	0.1443	0.034*	
H32B	0.138	0.0318	0.2672	0.034*	
H32C	0.0049	−0.065	0.1684	0.034*	
C1	0.40786 (14)	0.37152 (15)	0.65233 (14)	0.0127 (3)	
C12	0.30930 (15)	0.14154 (15)	0.69355 (15)	0.0143 (3)	
C15	0.32315 (16)	0.30349 (17)	0.96407 (16)	0.0183 (3)	
H15	0.3285	0.3569	1.0533	0.022*	
C2	0.29274 (14)	0.57566 (15)	0.47500 (14)	0.0119 (3)	
C13	0.26600 (15)	0.07881 (16)	0.78583 (16)	0.0166 (3)	
H13	0.2318	−0.0191	0.7571	0.02*	
C32	0.17142 (16)	−0.09665 (16)	0.09292 (15)	0.0168 (3)	
C21	0.17807 (14)	0.62904 (14)	0.45946 (15)	0.0131 (3)	
C33	0.10027 (17)	−0.22517 (17)	−0.00704 (16)	0.0229 (3)	
H33	0.0071	−0.2536	−0.0213	0.027*	
C22	0.13237 (16)	0.63473 (16)	0.33772 (16)	0.0182 (3)	
C121	0.30291 (18)	0.04573 (16)	0.55228 (16)	0.0211 (3)	
H12A	0.278	−0.0505	0.5475	0.032*	
H12B	0.3892	0.0707	0.5256	0.032*	
H12C	0.2375	0.0561	0.4939	0.032*	
C26	0.11778 (16)	0.67358 (16)	0.57076 (16)	0.0175 (3)	
H26	0.151	0.6714	0.6516	0.021*	
C34	0.16490 (19)	−0.31154 (17)	−0.08566 (16)	0.0254 (4)	
H34	0.1149	−0.396	−0.1525	0.03*	
C3	0.39545 (15)	0.08020 (15)	0.21105 (14)	0.0137 (3)	
C35	0.30350 (19)	−0.27297 (17)	−0.06539 (16)	0.0238 (4)	
H35	0.3469	−0.3305	−0.1189	0.029*	
C14	0.27235 (15)	0.15762 (17)	0.91873 (16)	0.0177 (3)	
H14	0.2425	0.1125	0.9775	0.021*	
C24	−0.03711 (16)	0.72774 (17)	0.44262 (19)	0.0261 (4)	
H24	−0.11	0.7598	0.436	0.031*	
C221	0.1966 (2)	0.5912 (2)	0.21626 (17)	0.0314 (4)	
H22A	0.2917	0.6378	0.2388	0.047*	
H22B	0.1603	0.6173	0.1481	0.047*	
H22C	0.1789	0.4909	0.1842	0.047*	
C23	0.02467 (17)	0.68697 (17)	0.33293 (18)	0.0250 (4)	
H23	−0.0062	0.6945	0.2539	0.03*	

### Atomic displacement parameters (Å^2^)

**Table:**

	*U*^11^	*U*^22^	*U*^33^	*U*^12^	*U*^13^	*U*^23^
Cu1	0.01089 (9)	0.00896 (9)	0.01017 (9)	0.00293 (6)	0.00121 (6)	0.00270 (6)
O32	0.0162 (5)	0.0158 (5)	0.0258 (6)	0.0068 (4)	−0.0053 (5)	−0.0019 (5)
O31	0.0165 (5)	0.0124 (5)	0.0150 (5)	0.0054 (4)	−0.0001 (4)	0.0019 (4)
O21	0.0211 (6)	0.0135 (5)	0.0143 (5)	0.0004 (4)	0.0038 (4)	0.0047 (4)
O22	0.0126 (5)	0.0159 (5)	0.0178 (5)	0.0056 (4)	−0.0018 (4)	0.0006 (4)
O11	0.0205 (5)	0.0135 (5)	0.0122 (5)	0.0031 (4)	0.0019 (4)	0.0053 (4)
O12	0.0145 (5)	0.0132 (5)	0.0157 (5)	0.0058 (4)	−0.0003 (4)	0.0025 (4)
C16	0.0170 (7)	0.0144 (7)	0.0160 (7)	0.0042 (6)	0.0020 (6)	0.0052 (6)
C31	0.0177 (7)	0.0117 (6)	0.0126 (7)	0.0041 (6)	0.0018 (6)	0.0043 (5)
C11	0.0099 (6)	0.0153 (7)	0.0139 (7)	0.0045 (5)	0.0023 (5)	0.0066 (6)
C36	0.0235 (8)	0.0156 (7)	0.0187 (8)	0.0083 (6)	0.0057 (6)	0.0057 (6)
C25	0.0163 (8)	0.0180 (8)	0.0319 (9)	0.0058 (6)	0.0088 (7)	0.0026 (7)
C321	0.0146 (7)	0.0262 (8)	0.0265 (9)	0.0060 (6)	0.0035 (6)	0.0082 (7)
C1	0.0115 (7)	0.0141 (7)	0.0142 (7)	0.0061 (5)	0.0027 (5)	0.0055 (5)
C12	0.0121 (7)	0.0154 (7)	0.0158 (7)	0.0048 (6)	0.0011 (5)	0.0057 (6)
C15	0.0197 (8)	0.0227 (8)	0.0139 (7)	0.0081 (6)	0.0042 (6)	0.0067 (6)
C2	0.0121 (7)	0.0116 (6)	0.0131 (7)	0.0026 (5)	0.0035 (5)	0.0066 (5)
C13	0.0134 (7)	0.0149 (7)	0.0216 (8)	0.0028 (6)	0.0021 (6)	0.0083 (6)
C32	0.0190 (7)	0.0156 (7)	0.0152 (7)	0.0028 (6)	0.0008 (6)	0.0073 (6)
C21	0.0109 (6)	0.0093 (6)	0.0169 (7)	0.0015 (5)	0.0013 (5)	0.0038 (5)
C33	0.0232 (8)	0.0182 (8)	0.0211 (8)	−0.0025 (6)	−0.0041 (7)	0.0082 (6)
C22	0.0196 (8)	0.0158 (7)	0.0181 (8)	0.0072 (6)	−0.0005 (6)	0.0038 (6)
C121	0.0297 (9)	0.0130 (7)	0.0189 (8)	0.0054 (6)	0.0039 (7)	0.0048 (6)
C26	0.0165 (7)	0.0151 (7)	0.0191 (7)	0.0040 (6)	0.0038 (6)	0.0044 (6)
C34	0.0394 (10)	0.0137 (7)	0.0149 (8)	−0.0004 (7)	−0.0029 (7)	0.0034 (6)
C3	0.0138 (7)	0.0146 (7)	0.0120 (7)	0.0041 (6)	0.0036 (5)	0.0043 (5)
C35	0.0401 (10)	0.0146 (7)	0.0167 (8)	0.0110 (7)	0.0069 (7)	0.0034 (6)
C14	0.0147 (7)	0.0242 (8)	0.0194 (8)	0.0063 (6)	0.0046 (6)	0.0141 (6)
C24	0.0141 (7)	0.0183 (8)	0.0399 (10)	0.0076 (6)	−0.0033 (7)	0.0022 (7)
C221	0.0442 (11)	0.0427 (11)	0.0175 (8)	0.0258 (9)	0.0062 (8)	0.0134 (8)
C23	0.0246 (9)	0.0205 (8)	0.0264 (9)	0.0094 (7)	−0.0085 (7)	0.0041 (7)

### Geometric parameters (Å, °)

**Table:**

Cu1—O21^i^	1.9402 (12)	C321—H32B	0.96
Cu1—O11	1.9559 (12)	C321—H32C	0.96
Cu1—O12	1.9585 (13)	C12—C13	1.397 (2)
Cu1—O22^i^	1.9900 (13)	C12—C121	1.510 (2)
Cu1—O31	2.1622 (13)	C15—C14	1.385 (2)
Cu1—Cu1^i^	2.5780 (9)	C15—H15	0.93
O32—C3	1.3184 (19)	C2—C21	1.492 (2)
O32—H32	0.82	C13—C14	1.386 (2)
O31—C3	1.2250 (18)	C13—H13	0.93
O21—C1	1.2670 (18)	C32—C33	1.394 (2)
O21—Cu1^i^	1.9402 (12)	C21—C26	1.394 (2)
O22—C2	1.2764 (18)	C21—C22	1.398 (2)
O22—Cu1^i^	1.9900 (13)	C33—C34	1.385 (3)
O11—C1	1.2626 (18)	C33—H33	0.93
O12—C2	1.2518 (18)	C22—C23	1.399 (2)
C16—C15	1.382 (2)	C22—C221	1.502 (2)
C16—C11	1.400 (2)	C121—H12A	0.96
C16—H16	0.93	C121—H12B	0.96
C31—C36	1.397 (2)	C121—H12C	0.96
C31—C32	1.404 (2)	C26—H26	0.93
C31—C3	1.485 (2)	C34—C35	1.384 (3)
C11—C12	1.410 (2)	C34—H34	0.93
C11—C1	1.497 (2)	C35—H35	0.93
C36—C35	1.384 (2)	C14—H14	0.93
C36—H36	0.93	C24—C23	1.383 (3)
C25—C24	1.381 (3)	C24—H24	0.93
C25—C26	1.383 (2)	C221—H22A	0.96
C25—H25	0.93	C221—H22B	0.96
C321—C32	1.506 (2)	C221—H22C	0.96
C321—H32A	0.96	C23—H23	0.93
			
O21^i^—Cu1—O11	170.01 (4)	O12—C2—O22	124.23 (14)
O21^i^—Cu1—O12	87.99 (5)	O12—C2—C21	118.96 (13)
O11—Cu1—O12	91.79 (5)	O22—C2—C21	116.81 (13)
O21^i^—Cu1—O22^i^	89.93 (5)	C14—C13—C12	122.17 (14)
O11—Cu1—O22^i^	88.55 (5)	C14—C13—H13	118.9
O12—Cu1—O22^i^	169.88 (4)	C12—C13—H13	118.9
O21^i^—Cu1—O31	98.74 (6)	C33—C32—C31	117.35 (15)
O11—Cu1—O31	91.16 (6)	C33—C32—C321	119.26 (15)
O12—Cu1—O31	99.14 (5)	C31—C32—C321	123.37 (14)
O22^i^—Cu1—O31	90.97 (5)	C26—C21—C22	120.83 (14)
O21^i^—Cu1—Cu1^i^	87.75 (5)	C26—C21—C2	117.76 (13)
O11—Cu1—Cu1^i^	82.27 (5)	C22—C21—C2	121.41 (13)
O12—Cu1—Cu1^i^	86.61 (4)	C34—C33—C32	121.67 (16)
O22^i^—Cu1—Cu1^i^	83.41 (4)	C34—C33—H33	119.2
O31—Cu1—Cu1^i^	171.44 (3)	C32—C33—H33	119.2
C3—O32—H32	109.5	C21—C22—C23	117.30 (15)
C3—O31—Cu1	129.50 (10)	C21—C22—C221	122.57 (15)
C1—O21—Cu1^i^	119.83 (10)	C23—C22—C221	120.12 (15)
C2—O22—Cu1^i^	123.41 (10)	C12—C121—H12A	109.5
C1—O11—Cu1	125.45 (10)	C12—C121—H12B	109.5
C2—O12—Cu1	122.02 (10)	H12A—C121—H12B	109.5
C15—C16—C11	121.39 (14)	C12—C121—H12C	109.5
C15—C16—H16	119.3	H12A—C121—H12C	109.5
C11—C16—H16	119.3	H12B—C121—H12C	109.5
C36—C31—C32	120.86 (14)	C25—C26—C21	120.55 (15)
C36—C31—C3	118.27 (14)	C25—C26—H26	119.7
C32—C31—C3	120.78 (13)	C21—C26—H26	119.7
C16—C11—C12	119.90 (13)	C35—C34—C33	120.44 (15)
C16—C11—C1	116.94 (13)	C35—C34—H34	119.8
C12—C11—C1	123.16 (13)	C33—C34—H34	119.8
C35—C36—C31	120.42 (16)	O31—C3—O32	123.59 (13)
C35—C36—H36	119.8	O31—C3—C31	122.29 (14)
C31—C36—H36	119.8	O32—C3—C31	114.10 (13)
C24—C25—C26	119.35 (16)	C36—C35—C34	119.22 (16)
C24—C25—H25	120.3	C36—C35—H35	120.4
C26—C25—H25	120.3	C34—C35—H35	120.4
C32—C321—H32A	109.5	C15—C14—C13	120.05 (14)
C32—C321—H32B	109.5	C15—C14—H14	120
H32A—C321—H32B	109.5	C13—C14—H14	120
C32—C321—H32C	109.5	C25—C24—C23	120.20 (16)
H32A—C321—H32C	109.5	C25—C24—H24	119.9
H32B—C321—H32C	109.5	C23—C24—H24	119.9
O11—C1—O21	124.51 (13)	C22—C221—H22A	109.5
O11—C1—C11	118.61 (13)	C22—C221—H22B	109.5
O21—C1—C11	116.88 (13)	H22A—C221—H22B	109.5
C13—C12—C11	117.39 (14)	C22—C221—H22C	109.5
C13—C12—C121	117.89 (14)	H22A—C221—H22C	109.5
C11—C12—C121	124.70 (13)	H22B—C221—H22C	109.5
C16—C15—C14	119.09 (15)	C24—C23—C22	121.72 (16)
C16—C15—H15	120.5	C24—C23—H23	119.1
C14—C15—H15	120.5	C22—C23—H23	119.1
			
O21^i^—Cu1—O31—C3	108.89 (13)	C11—C12—C13—C14	−0.8 (2)
O11—Cu1—O31—C3	−69.75 (13)	C121—C12—C13—C14	177.70 (14)
O12—Cu1—O31—C3	−161.75 (13)	C36—C31—C32—C33	−0.5 (2)
O22^i^—Cu1—O31—C3	18.81 (13)	C3—C31—C32—C33	176.08 (14)
O12—Cu1—O11—C1	−88.41 (12)	C36—C31—C32—C321	178.06 (14)
O22^i^—Cu1—O11—C1	81.47 (12)	C3—C31—C32—C321	−5.4 (2)
O31—Cu1—O11—C1	172.41 (12)	O12—C2—C21—C26	131.59 (15)
Cu1^i^—Cu1—O11—C1	−2.08 (11)	O22—C2—C21—C26	−47.95 (19)
O21^i^—Cu1—O12—C2	−87.01 (12)	O12—C2—C21—C22	−48.5 (2)
O11—Cu1—O12—C2	83.00 (12)	O22—C2—C21—C22	131.91 (15)
O22^i^—Cu1—O12—C2	−8.8 (3)	C31—C32—C33—C34	−1.1 (2)
O31—Cu1—O12—C2	174.46 (11)	C321—C32—C33—C34	−179.70 (15)
Cu1^i^—Cu1—O12—C2	0.85 (11)	C26—C21—C22—C23	0.1 (2)
C15—C16—C11—C12	−0.8 (2)	C2—C21—C22—C23	−179.71 (14)
C15—C16—C11—C1	179.95 (14)	C26—C21—C22—C221	178.43 (16)
C32—C31—C36—C35	2.2 (2)	C2—C21—C22—C221	−1.4 (2)
C3—C31—C36—C35	−174.48 (14)	C24—C25—C26—C21	−1.9 (2)
Cu1—O11—C1—O21	5.1 (2)	C22—C21—C26—C25	1.7 (2)
Cu1—O11—C1—C11	−175.02 (9)	C2—C21—C26—C25	−178.42 (14)
Cu1^i^—O21—C1—O11	−5.4 (2)	C32—C33—C34—C35	1.0 (2)
Cu1^i^—O21—C1—C11	174.78 (9)	Cu1—O31—C3—O32	−13.1 (2)
C16—C11—C1—O11	−166.58 (13)	Cu1—O31—C3—C31	168.70 (10)
C12—C11—C1—O11	14.2 (2)	C36—C31—C3—O31	144.89 (15)
C16—C11—C1—O21	13.27 (19)	C32—C31—C3—O31	−31.8 (2)
C12—C11—C1—O21	−165.91 (14)	C36—C31—C3—O32	−33.43 (19)
C16—C11—C12—C13	1.3 (2)	C32—C31—C3—O32	149.91 (14)
C1—C11—C12—C13	−179.57 (13)	C31—C36—C35—C34	−2.2 (2)
C16—C11—C12—C121	−177.11 (14)	C33—C34—C35—C36	0.7 (2)
C1—C11—C12—C121	2.1 (2)	C16—C15—C14—C13	0.6 (2)
C11—C16—C15—C14	−0.1 (2)	C12—C13—C14—C15	−0.2 (2)
Cu1—O12—C2—O22	−5.0 (2)	C26—C25—C24—C23	0.3 (2)
Cu1—O12—C2—C21	175.52 (9)	C25—C24—C23—C22	1.6 (3)
Cu1^i^—O22—C2—O12	7.4 (2)	C21—C22—C23—C24	−1.8 (2)
Cu1^i^—O22—C2—C21	−173.12 (9)	C221—C22—C23—C24	179.87 (17)

Symmetry codes: (i) −*x*+1, −*y*+1, −*z*+1.

### Hydrogen-bond geometry (Å, °)

**Table:**

*D*—H···*A*	*D*—H	H···*A*	*D*···*A*	*D*—H···*A*
O32—H32···O22^i^	0.82	1.85	2.6604 (18)	168
C16—H16···O21	0.93	2.39	2.721 (2)	101

Symmetry codes: (i) −*x*+1, −*y*+1, −*z*+1.
